# The association between cholecystectomy and colorectal neoplasm in inflammatory bowel diseases: A population-based cohort study

**DOI:** 10.1371/journal.pone.0177745

**Published:** 2017-05-26

**Authors:** Yen-Chun Peng, Cheng-Li Lin, Fung-Chang Sung

**Affiliations:** 1 Division of Gastroenterology, Department of Internal Medicine, Taichung Veterans General Hospital, Taichung, Taiwan; 2 National Yang-Ming University, Taipei, Taiwan; 3 Management Office for Health Data, China Medical University Hospital, Taichung, Taiwan; 4 Graduate Institute of Clinical Medical Science, College of Medicine, China Medical University, Taichung, Taiwan; 5 Department of Health Services Administration, College of Public Health, China Medical University, Taichung, Taiwan; University Hospital Llandough, UNITED KINGDOM

## Abstract

**Background & aims:**

Inflammatory bowel diseases (IBD) and cholecystectomy are associated with the risk of colorectal cancer (CRC). Our aim was to determine the association between cholecystectomy and the CRC risk in IBD.

**Methods:**

We first obtained the Taiwan National Health Insurance Research Database (NHRID), which contains information on approximately 24.7 million insured individuals. A cohort study was conducted using the data from the NHIRD, and included cohort patients with IBD who had experienced a cholecystectomy between the years 1998 and 2010. The non-cholecystectomy cohort comprised the remaining IBD patients who had not undergone a cholecystectomy. Multivariate Cox proportional hazard regression analysis was used to determine the effects cholecystectomy have on the risks of developing CRC, as shown by Hazard Ratios (HRs) with 95% confidence intervals (CIs).

**Results:**

The incidence rate of CRC among IBD patients who had undergone a cholecystectomy (n = 525) was 1.75 per 1,000 person-years, compared to 1.41 per 1,000 person-years among IBD patients who had not had a cholecystectomy (n = 525). The adjusted HRs for CRC was found to be 0.76 (95% CI 0.25–2.32) for IBD patients having received a cholecystectomy, after adjusting for age, gender, and comorbidities. By type of IBD, neither ulcerative colitis nor Crohn’s diseases are associated with CRC after a cholecystectomy adjusted HR (2.78 [95% CI 0.54–14.3]) and (0.13 [95% CI 0.01–1.49]).

**Conclusion:**

In Taiwan, cholecystectomies are not associated with a risk of CRC in patients with IBD.

## Introduction

A recent systemic analysis has concluded that colorectal cancer (CRC) remains a problem in patients with inflammatory bowel disease (IBD). [1] Time-trend studies also demonstrated a decreasing risk of CRC in ulcerative colitis patients. [2] Cholecystectomy is also considered a risk associated with developing intestinal cancers, including CRC.[3–7] By location, this positive association is strictly for colon cancer, and not for rectal cancer. [4] Cholecystectomy in recurrent adenomas and multiple advanced recurrent adenomas are considered to present a slightly increased risk of CRC. [5] However, some studies failed to demonstrate an association between cholecystectomy and intestinal cancers. [8,9] Additionally, performing a cholecystectomy via laparoscopy or other surgical technique has not yet been investigated for carrying different risks for CRC.

The risk of CRC after a cholecystectomy has been defined in several studies for the general population.[3–5,7,10] The risk of intestinal cancers declines with the increase of distance from the common bile duct after a cholecystectomy. The cancer risk of the rectum is also not associated with a cholecystectomy. [4] IBD is a known prevalent intestine disorder. IBD patients may experience a cholecystectomy. No study has investigated whether cholecystectomy alter the risk of CRC. This study aimed to evaluate the risk of CRC after a cholecystectomy in patients with IBD using claims data obtained from the National Health Insurance (NHI) program of Taiwan.

## Materials and methods

### Data source

The NHI program was established in 1995 with 99% of the 23.74 million residents of Taiwan compulsively covered. In cooperation with the Bureau of NHI, the National Health Research Institutes of Taiwan randomly sampled a representative database from all NHI enrollees, using a systematic sampling method for research purposes, known as the National Health Research Institute Database (NHRID). Diseases are coded with International Classification of Diseases, Ninth revision, Clinical Modification (ICD-9-CM). This study was approved by the Research Ethic Committee, China Medical University and Hospital in Taiwan (CMUH104-REC2-115).

### Study design and sampled patients

We conducted a retrospective cohort study using the NHIRD of the NHI program to identify IBD cases (ICD-9-CM codes 555–556) from inpatient records in years 1998–2010 and patients, aged 20 or older were include. The dates of patient’s first hospitalization for IBD were defined as the index date, the starting follow-up time. During follow-up period, IBD patients with the history of cholecystectomy (ICD-9-OP 51.22, 51.23) were considered as the cholecystectomy cohort. We excluded patients with the history of CRC (ICD-9-CM code 153, 154) before the index date, or with any CRC diagnosed within 1 year after the index date. Patients younger than 20 years or without information of age and sex were also excluded. The non-cholecystectomy cohort was also identified from data of IBD patients diagnosed in 1998–2010 without the history of CRC and cholecystectomy. For each cholecystectomy patient in the study cohort, one non-cholecystectomy patient was frequency-matched by the year of the diagnosis date.

### Outcome

The follow-up time estimation for each patient was started on the date at IBD diagnosis for both cohorts. They were monitored until a diagnosis of CRC (ICD-9 code 153–154) was made, were censored for withdrawal from the NHI program, death, or until the date of December 31, 2011, whichever occurred first. The diagnosis of CRC was confirmed by the Registry for Catastrophic Illness Patient Database (RCIPD), which is a sub-dataset of the NHIRD. The “catastrophic illness certificate” application requires an additional review of medical reports, image, laboratory data, and pathology findings by experts.

### Statistical analysis

Distributions of demographic variables (age and gender) and comorbidities were compared between cohorts with and without a cholecystectomy in IBD patients. The Chi-square test and Student’s *t* test were used to examine categorical variable and continuous variables, respectively. Incidence rate density of CRC by gender, age, and comorbidity were estimated for both cohorts. The cumulative incidence rates of CRC using the Kaplan-Meier method in both cohorts were plotted and examined by the log-rank test. Univariable and multivariable Cox proportional hazards regression analyses were performed to estimate the crude hazard ratios (cHRs) and adjusted hazard ratios (aHRs), respectively, along with 95% Confidence Intervals (CIs). The multivariable models were simultaneously adjusted for age, sex, and the comorbidities of hypertension, diabetes, hyperlipidemia, stroke, congestive heart failure, colorectal adenomas and obesity. All data processing and statistical analyses were performed using SAS Version 9.4 (SAS Institute, Inc., Cary, NC, USA). A two-tailed p < .05 was considered significant.

## Results

### Demographic and comorbidity data

There were 525 patients who had undergone a cholecystectomy, and 525 patients who had not, in the IBD patients (Table 1). IBD patients receiving cholecystectomy were approximately 10 years older than those without a cholecystectomy (p <0.001). The follow-up durations were 7.63 ±3.94 and 8.09 ±3.87 in cohorts with and without cholecystectomy, respectively. The cholecystectomy cohort was more prevalent with most comorbidities, including hypertension (32.4% vs. 16.4%, p<0.001), diabetes (20.0% vs. 8.0%, p<0.001), hyperlipidemia (9.90% vs. 7.05%, p<0.10), stroke (9.52% vs. 6.67%), congestive heart failure (5.71% vs. 2.10%, p<0.003) and colorectal adenoma (3.43% vs. 1.14%, p<0.01), except obesity (0.19% vs. 0.19%, p = 0.99).

**Table 1:** Comparison of demographics and comorbidity among inflammatory bowel disease patients with cholecystectomyand without cholecystectomy.

	Cholecystectomy		p-value
	No(N = 525)	Yes(N = 525)	
	n(%)	n(%)	
Age, year			<0.001
≤ 49	287(54.7)	153(29.1)	
50–64	96(18.3)	149(28.4)	
≧65	142(27.1)	223(42.5)	
Mean (SD)^†^	49.2(19.4)	59.0(16.0)	<0.001
Follow-up time (SD)^†^	8.09(3.87)	7.63(3.94)	0.06
Gender			0.17
Female	236(45.0)	258(49.1)	
Male	289(55.1)	267(50.9)	
Comorbidity			
Hypertension	86(16.4)	170(32.4)	<0.001
Diabetes	42(8.00)	107(20.4)	<0.001
Hyperlipidemia	37(7.05)	52(9.90)	0.1
Stroke	35(6.67)	50(9.52)	0.09
Congestive heart failure	11(2.10)	30(5.71)	0.003
Colorectal adenomas	6(1.14)	18(3.43)	0.01
Obesity	1(0.19)	1(0.19)	0.99

Chi-square test

†T-test

### Incident CRC by age, gender and comorbidity

**Fig 1:**
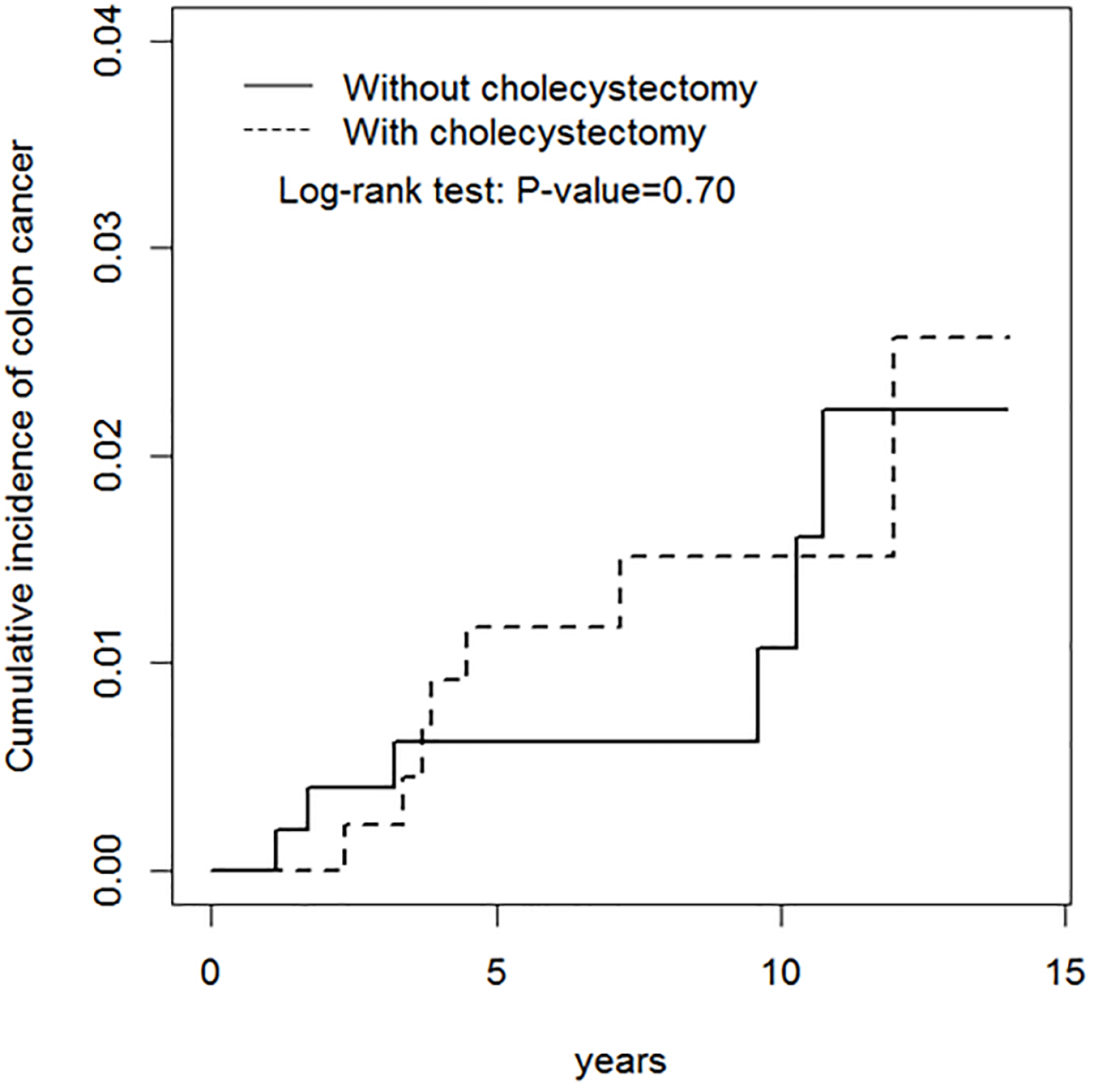
Kaplan-Meir method determined cumulative incidence of colorectal cancer compared between cholecystectomy cohort and non-cholecystectomy cohort.

Fig 1 shows the cumulative incidence rates of CRC for cohorts with and without cholecystectomy in IBD patients. There was no significant difference (log-rank test P = 0.70).

The overall incidence of CRC was slightly greater inthe cholecystectomy cohort than in the non-cholecystectomy cohort (1.75 vs 1.41, respectively, per 1000 person-y) with an aHR of 0.76 (95% CI = 0.25–2.32) (Table 2). The stratified analysis showed that the CRC risks were not associated with gender, age and comorbidity.

**Table 2:** Incidence and adjusted hazard ratio of colorectal cancer stratified by sex, age and comorbidity compared among inflammatory bowel disease patients with cholecystectomy and without cholecystectomy.

	Cholecystectomy							
	No			Yes				
Variables	No. of Events	PY	Rate^#^	No. of Events	PY	Rate^#^	Crude HR (95%CI)	Adjusted HR (95%CI)†
All	6	4245	1.41	7	4006	1.75	1.24(0.42, 3.68)	0.76(0.25, 2.32)
Gender								
Female	1	1933	0.52	3	2025	1.48	2.84(0.30, 27.3)	1.62(0.16, 16.4)
Male	5	2312	2.16	4	1981	2.02	0.94(0.25, 3.51)	0.57(0.14, 2.22)
Age								
≤ 49	2	2421	0.83	0	1319	0	-	-
50–64	1	833	1.2	3	1233	2.43	2.07(0.22, 19.9)	1.74(0.18, 16.9)
≧65	3	991	3.03	4	1454	2.75	0.92(0.21, 4.10)	0.74(0.16, 3.37)
Comorbidity^‡^								
No	3	3430	0.87	4	2345	1.71	1.91(0.43, 8.54)	1.43(0.32, 6.47)
Yes	3	816	3.68	3	1661	1.81	0.49(0.10, 2.44)	0.52(0.10, 2.56)

Rate#, incidence rate, per 1000 person-years; Crude HR, relative hazard ratio

Adjusted HR†: multivariable analysis including age, sex, and comorbidities

Comorbidity‡: Patients with any one of the comorbidities hypertension, diabetes, hyperlipidemia, stroke, congestive heart failure, colorectal adenomas, and obesity were classified as the comorbidity group

### Incident CRC associated with ulcerative colitis and Crohn’s disease

Table 3 shows the incident CRC in IBD patients with ulcerative colitis was greater in those with cholecystectomy than those without cholecystectomy, with an aHR of 2.78 (95% CI 0.54–14.3). On the other hand, ulcerative colitis was associated with a reduced CRC incidence in the cholecystectomy cohort with an aHR of 0.13 (95% CI 0.01–1.49).

**Table 3:** Incidence and adjusted hazard ratio of colorectal cancer stratified by different entities of inflammatory bowel disease patients with or without cholecystectomy.

	Cholecystectomy									
	No				Yes					
Variables	N	No. of Events	PY	Rate^#^	N	No. of Events	PY	Rate^#^	Crude HR	Adjusted HR
									(95%CI)	(95%CI) †
Ulcerative colitis	234	2	1897	1.05	214	6	1533	3.91	3.53	2.78
									(0.71, 17.4)	(0.54, 14.3)
	
Crohn’s disease	291	4	2349	1.7	311	1	2473	0.4	0.24	0.13
									(0.03, 2.18)	(0.01, 1.49)
	

Rate#, incidence rate, per 1000 person-years; Crude HR, relative hazard ratio

Adjusted HR†: multivariable analysis including age, sex, and comorbidities

### CRC risk by cancer site and type of cholecystectomy

Incidence and aHR of CRC by the cancer site and type of cholecystectomy are demonstrated in Table 4. The CRC incidence in IBD patients was 6.6-fold greater in those with non-laparoscopic cholecystectomy than those with laparoscopic cholecystectomy (2.90 vs. 0.44 per 1000 person-years).

**Table 4:** Incidence of colorectal cancer location using Cox model measured hazards ratio by type of cholecystectomy.

Variables(ICD-9 code)	Event	Rate^#^	Crude HR*	Adjusted HR^†^ (95% CI)
			(95% CI)	
Colon cancer				
Non-cholecystectomycohort (N = 525)	6	1.41	1(Reference)	1(Reference)
Cholecystectomy				
Lapraroscopic cholecystectomy (N = 282)	2	0.88	0.62(0.13, 3.07)	0.44(0.09, 2.22)
Non-lapraroscopic cholecystectomy (N = 243)	5	2.9	2.06(0.63, 6.76)	1.11(0.32, 3.78)
Colon cancer (except rectum) (ICD-9 153)				
Non-cholecystectomycohort (N = 525)	3	0.71	1(Reference)	1(Reference)
Cholecystectomy				
Lapraroscopic cholecystectomy (N = 282)	1	0.44	0.62(0.07, 5.99)	0.40(0.04, 3.92)
Non-lapraroscopic cholecystectomy (N = 243)	5	2.9	4.21(1.01, 17.6)*	2.81(0.63, 12.5)
Rectal cancer (ICD-9 154)				
Non-cholecystectomycohort (N = 525)	3	0.71	1(Reference)	1(Reference)
Cholecystectomy				
Lapraroscopic cholecystectomy (N = 282)	1	0.44	0.61(0.06, 5.87)	0.42(0.04, 4.80)
Non-lapraroscopic cholecystectomy (N = 243)	0	0	-	-

Rate#, incidence rate, per 1,000 person-years; Crude HR *, relative hazard ratio; Adjusted HR†: multivariable analysis including age, sex, and comorbidities of hyperlipidemia, diabetes, liver cirrhosis, hypertension, COPD, stroke, CAD, and HCV

## Discussion

This study failed to demonstrate an association between cholecystectomies and CRC risk for IBD patients. The incidence rate of CRC was somewhat higher for those with cholecystectomy than those without the cholecystectomy. However, the aHR of CRC was 0.76 (95% CI 0.25–2.32) for IBD patients receiving a cholecystectomy, after adjusting for age, sex, and comorbidities. In Taiwan, therefor, a cholecystectomy would not be associated with CRC among IBD patients. By type of IBD, neither ulcerative colitis nor Crohn’s disease is associated with CRC after a cholecystectomy. By type of cholecystectomy, a laparoscopic or non-laparoscopic cholecystectomy was not associated with CRC in IBD patients. By location, both colon and rectal cancer patients are not at a risk of CRC in IBD.

Throughout the general population, a cholecystectomy is considered to pose a CRC risk for CRC, as shown in several studies.[3–5,7,10] However, other studies failed to show that a cholecystectomy carry a risk of either CRC or gastrointestinal cancer.[8,9,11–14] In the IBD population, patients with IBD are associated with an increased risk of cholelithiasis, with similar inflammatory patterns in cholecystectomy specimens, when compared with the general population. [15] Previous studies have reported that both IBD patients and cholecystectomy patients had increased risk of CRCs. We, thus, hypothesized that IBD patients undergone a cholecystectomy would be at an increased risk of CRC. A meta-analysis has reported that the prevalence of choledocholithiasis tended to be increased in IBD patients. [16] The risk of CRC after a cholecystectomy in IBD patients has not been addressed. The present study was the first one for evaluating the risk of CRC in IBD patients who had received a cholecystectomy, and found no significant association in Taiwanese patients.

Several factors, such as duration and extension of colitis, onset of age, and family history of CRC, have been identified as factors that may increase or decrease the CRC risk in patients with IBD. [17] Primary sclerosing cholangitis is considered as a biliary risk factor for CRC in IBD. Focusing on the issue of increased incidence of cholelithiasis, a cholecystectomy would be investigated for the possible association of CRC in IBD.

Schernhammer et al. have reported a significant cancer risk in the proximal colon and the rectum for patients with cholecystectomy. [3] The risk decreases as the distance from the biliary duct increases. [4] In IBD patients, our results demonstrated that the rectum is not at a higher CRC risk in IBD patients.

IBD patients undergoing cholecystectomy were shown to have a significantly increased cancer risk for postoperative complications. [18] There should be concern regarding the risks and complications of surgery in patients with IBD. Laparoscopic cholecystectomies have been convincingly shown to be superior to open cholecystectomies on the basis of controlled clinical trial results. [19] Several factors should be considered before undergoing a laparoscopic cholecystectomy, such as the timing, a complicated cholecystitis, and any host factors. Our data did show a greater CRC incidence in IBD patients with non-laparoscopic cholecystectomy than those with laparoscopic cholecystectomy.

There are limitations in the present study. First, though IBD patients are at risk of cholelithiasis, the patient numbers are still limited in this population-based study. The study sample was limited with a small size of 525 in each cohort and with only 7 CRC events in the cholecystectomy cohort and 6 CRC events in the non-cholecystectomy cohort. This study is underpowered. Second, IBD carries a relatively lower incidence rate in Taiwan, as well as in the rest of Asia. Therefore, any ethnic factors should be considered. A study composed of people from both the Eastern and Western world needs to be conducted to achieve a sounder conclusion.

In conclusion, cholecystectomies do not carry a cancer risk in the colon and rectum among IBD patients. A cholecystectomy using laparoscopic or non-laparoscopic surgery also does not affect risk for CRC in the IBD patient population.

## Abbreviations

HRs: hazard ratios
CIs: confidence intervals
CRC: colorectal cancer
IBD: inflammatory bowel diseases
NHI: National Health Insurance
NHIRD: National Health Research Institute Database
